# Notes on Some Cases of Malarial Diseases

**Published:** 1881-11

**Authors:** A. B. Arnold

**Affiliations:** Professor of Clinical Medicine and Diseases of the Nervous System, College of Physicians and Surgeons, Baltimore


ARTICLE II.

### NOTES ON SOME CASES OF MALARIAL DISEASE.

BY A. B. ARNOLD, M. D.,

Professor of Clinical Medicine and Diseases of the Nervous System, College of Physicians and Surgeons, Baltimore.

[Read before the Balto. Med. and Surg. Society.]

Miasmatic fevers of a remittent type prevail to an unusual extent in our city and its environs at the present time, which correspond in every particular to the excellent descriptions of the older American writers on indigenous febrile diseases. I would especially allude to the monographs of Smith, Gerhard, Stewardson, Yandell and Bartlett. The cases which came under my notice during the latter half of August, and throughout the month of September, presented only one peculiarity, namely :—the frequent absence of the initial chill. I do not here include that form of remittent or sub-continuous fever, which develops from a regular intermittent, whether quotidian or tertian. This failure of the ordinary chill or shiverings hardly embarrasses diagnosis, if the pyrexia shows decided remissions. But these were often missed, especially in hospital patients, who, as a class, are apt to present the adynamic condition. Some of these cases had also diarrhea, and thus an assemblage of symptoms would be established that might readily suggest the existence of the typhoid element. None of the cases, however, that came under my notice, answered to the description of the so-called typho-malarial species of fever. Even severe examples of the remittent type, which nearly partook of the continuous character, could be easily distinguished from the true typhoid.

The ordinary remittent that I observed, had seldom a prodromic stage. The headache was usually intense and prolonged ; the pain frequently located in the occipital region, and the aching sensation in the back and the limbs entirely differed from the feeling of tiredness and nervous prostration, so characteristic of enteric fever. Gastric and bilious disturbances being exceedingly common and persistent, justifies the old appellation : "bilious remittent fever." A disagreeable sensation of fullness and oppression in the epigastrium, extending also to both hypochondriac regions, was well marked in my cases.

Although the curative effect of the anti-periodic remedy is a capital test of all forms of malarial infection, yet it is a common observation that in severe cases of remittent fever, its action is not as prompt and satisfactory as might be expected. Perhaps the most important diagnostic sign in doubtful cases is the bodily temperature, which reaches its maximum at the very onset of the febrile paroxysm, and as suddenly falls, if only for a short time, during the course of twenty-four hours. The enlargement of the spleen when occurring early in the fever, is also of diagnostic value.

The treatment of bilious remittent fever at the present day shows no improvement on that which was recommended and adopted by the older physicians. Nor is it easy to conceive how it could be otherwise, since the introduction of cinchona and its preparations, into practice, which, after all, do the real work however variously combined with other drugs. My experience does not permit me to share the disparagement cast upon the practice of prescribing an occasional dose of the mercurial at least in the remittent variety, which is so frequently accompanied by severe gastric and biliary symptoms.

Without wishing to enter into a discussion whether these complications require any separate treatment, or whether blue-mass exercises any direct therapeutical influences upon the functions of the liver, it may be remarked, that to ignore the experience of many eminent medical men who adopt the practice, would greatly savor of an unjustifiable skepticism.

Like many others, I prefer the salts of soda and potash to combat the functional disturbances of the stomach and liver. Convalescence is sometimes rendered tedious on account of a weakened condition of the digestive powers. I have derived considerable advantage under these circumstances from chinoidinc:

| | | | |
|---|---|---|---|
| ℞ | Chinoidinae Pulv. . . . . . | grs. LXIV. |
| | Acid Tartar. . . . . . | grs. XVI. |
| | Glycerini . . . . . . | |
| | Alcohol āā . . . . . . | ℥ ss. |
| | Syr. Aurant. Cort. ad. . . . . . | ℥ iv. |

M. S. From a tea to a dessert spoonful before meals.

During the prevalence of the fall fevers of this year an unusual number of neuralgias came under my observation, which were, undoubtedly, examples of the masked intermittent. Singular enough, the branches of the trigeminus appear to be exempted, while by far the greater number of these neuralgias implicated the cervico-occipital, the intercostal, the brachial and the dorso-abdominal nerves. That these neuralgic affections were of malarial origin became evident from the prompt relief afforded by large doses of quinia. It is also worthy of notice that the exacerbation of pain was more marked in the morning hours than at any other part of the day.

Not many physicians, I venture to say, who practice in the latitude of Baltimore, come across cases of pernicious intermittent fever. The rarity of this species of malarial disease in this region of country is proved by the fact that upon inquiry among my professional brethren in this city, I found but few who had ever seen such a case. I do not remember that during an active practice extending over thirty (30) years, I had witnessed more than half a dozen cases of this terrible disease. Nor would it be surprising for reason of its rare occurrence, if it were mistaken for some kindred disease, especially at a season of the year when the diagnosis would probably turn upon some gastro-intestinal affection of exceptional violence.

I shall give the brief histories of two cases, illustrative of the choleraic variety of pernicious intermittent.

A middle-aged married woman living on the Belair road near the Schützen Park had been ill for two days. She was suddenly attacked with vomiting and a painless diarrhea that came on simultaneously with a moderate chill. These symptoms improved during the course of the day, but returned late in the evening with greater intensity. When I saw her next morning, she was evidently sinking. The shrunken features, sunken eyes, blue lips, coldness of surface, imperceptible pulse and embarrassed respiration, presented all the characteristics of the algid stage of Asiatic cholera. She lingered in this condition for nearly six hours, her mind remaining clear until a few moments before death took place. None of the measures I adopted to give her relief were of any avail. Large doses of quinia by the mouth and rectum were immediately rejected. The subcutaneous injection of this remedy did not then suggest itself to me, though I am convinced that the case was a hopeless one. The other case happened in the person of a young married lady, who, during the early part of August, had removed from the city to her country residence near the Waverly road. Her husband informed me that she had shivering fits on the day before accompanied by profuse, colorless discharges from the bowels. She apparently recovered from this attack, and on the following morning took breakfast with the family. I saw her for the first time at noon of the same day. As the weather was intensly hot, she had been placed on a temporary bed outside of the house. The patient complained of great thirst and nausea, of a sensation of oppression and constriction in the regions of the heart, difficulty of breathing and a feeling of utter exhaustion. She was wrapped in woolen blankets as she had occasional chills during the forenoon. I was informed by the nurse that the patient vomited everything she took into her stomach, and that involuntary discharges resembling dishwater incessantly passed from her bowels. It was almost impossible to keep her in bed on account of her restlessness and jactitation. Even a hasty examination of the patient's condition left no doubt in my mind that she was in imminent peril. Her widely open and injected eyes peered about from one object to another. There was an extremely anxious expression of her countenance, which was deeply cyanosed. The heart labored violently, and the pulse at the wrist was rapid, feeble and irregular. A clammy sweat covered the whole surface of the body. The coldness of the cheeks, ears, fingers and toes, contrasted strangely with the intense heat of the head and abdomen. Fearing the worst for my patient, I availed myself of the eminent services of Prof. McSherry, but before his arrival a decided change in the aspect of the case took place. A wild maniacal delirium suddenly set in and was quickly followed by fatal coma.

